# Efficacy and safety of streptokinase in prosthetic valve thrombosis (total 5 years clinical registry)

**DOI:** 10.4103/0253-7613.70403

**Published:** 2010-10

**Authors:** Kamal H. Sharma, Nishith M. Mewada

**Affiliations:** Department of Cardiology, U. N. Mehta Institute of Cardiology and Research Centre, Ahmedabad, India; 1 Life Care Institute of Medical Sciences and Research, Ahmedabad, India E-mail: nishith_mewada@hotmail.com

Sir,

Rheumatic heart disease is the most common cause of multivalvular disease in developing countries. Many of the patients need intervention in the form of valve replacement. The patients, who undergo replacement, carry high early and late morbidity rates because of primary valve failure, valve thrombosis, endocarditis, thromboembolism, and hemolytic anemia. Prosthetic valve thrombosis is one of the major causes of primary valve failure. It has been noted that with proper anticoagulation, the incidence rate of valve thrombosis is 0.1–5.7% per patient per year. Even with the use of warfarin, risk of thromboembolism is 1–2% per year, but the risk is considerably higher without treatment with warfarin.[1,2] Almost all studies have shown that the risk of embolism is greater with a valve in the mitral position (mechanical or biological) than with one in the aortic region.[1,2] High mortality rates with re-do surgery necessitate thrombolytic treatment with streptokinase followed by anticoagulation with unfractionated heparin and warfarin. There is very little data available about the effectiveness of streptokinase as a thrombolytic treatment. Failure to treat valve thrombosis carries a very high mortality rate,[3] and that is the reason why we have evaluated the safety and efficacy of streptokinase as the drug of choice.

The aim of the study was to evaluate the efficacy and safety of streptokinase as a thrombolytic therapy in prosthetic valve thrombosis and to evaluate the incidence rate of complications in a 1-month follow-up period.

Our study model was active-treatment concurrent control design where source of the data was the patients in clinical registry who had single valve involvement, who underwent prosthetic valve replacement surgery[3,4] and developed valvular thrombosis at U.N. Mehta Hospital and Life Care Institute of Medical Sciences and Research, Ahmedabad, India.

We retrieved and analyzed data of 48 patients who were diagnosed to have thrombosis of the prosthetic valve and treated with streptokinase. All the patients had a history of rheumatic heart disease, and all of them had undergone prosthetic valve replacement. All patients were on oral anticoagulant for prevention of valve thrombosis either in the form of warfarin or nicoumalone, and their INR at the time of valve thrombosis ranged from 1 to 3.2[5] Forty-four patients had mitral valve thrombosis, and four had aortic valve involvement. The clinical presentation was gradual onset breathlessness or acute pulmonary edema. Eight patients had NYHA-IV, 31 patients had NYHA-III, and 9 patients were NYHA-II status of dyspnea. All of them were thoroughly evaluated (2D Echo was the most important parameter) for the valve thrombosis. We included only those patients who had no contraindications to the thrombolytic therapy.

All patients were treated with the standard regimen of streptokinase infusion with 2.5 lac IU bolus, followed by 1 lac IU per hour for 48–72 h depending upon clinical and 2D Echo observations. Average duration of infusion of streptokinase was 52.2 h, and average total dose given was 54.7 lac IU.[1] Successful thrombolysis was defined as following:

## Total (Complete) Response

(1) Return of the transmitral gradient to baseline values if available or (2) Doubling of valve area from baseline thrombosis on 2D Echo and at least halving in the mean transmitral gradient post-streptokinase infusion.

## Partial Response

Intermediate reduction between total and halving in the mean transmitral gradient.

## Failed Response

No or minimal reduction in mean transmitral gradient.

All successfully thrombolysed patients were discharged on 75–150 mg of aspirin apart from anticoagulants in the form of warfarin or nicoumalone to maintain an INR between 2 and 2.5. Unfractionated heparin was started 12 h after the completion of streptokinase in a dose of 5000 IU IV 6 hourly and overlapped with oral anticoagulant. Heparin was continued till target INR was achieved with oral anticoagulant. Up to 1-month follow-up, all patients who had successful lysis of the thrombosed valve maintained the reduced gradient they had attained, and no complications were evident on follow-up.

We analyzed the data for incidence rate of various events and outcome after thrombolytic treatment with streptokinase. Out of 48 patients, 43 (90%) patients had successful thrombolysis as defined earlier. Total response was seen in 39 (81%) out of 43 patients, and 4 (8%) patients showed a partial response. Five patients (10%) had incomplete response (no or minimal reduction in transmitral gradient) and were classified as failed treatment. Three patients (6%) died during the hospital stay due to intracranial hemorrhage and heart failure. None of the patients showed any other major bleeding, central, or peripheral embolism during the treatment or up to 1-month of follow-up. We conclude that streptokinase is highly effective in the treatment of prosthetic valve thrombosis and has low incidence adverse events (6%) and therefore it should be considered as the first-line treatment for prosthetic valve thrombosis.[3,4]
